# Phytochemical Constituents and Antioxidant Activity of Sweet Basil (*Ocimum basilicum L*.) Essential Oil on Ground Beef from Boran and Nguni Cattle

**DOI:** 10.1155/2019/2628747

**Published:** 2019-01-01

**Authors:** Andrew Bamidele Falowo, Felicitas Esnart Mukumbo, Emrobowansan Monday Idamokoro, Anthony Jide Afolayan, Voster Muchenje

**Affiliations:** ^1^Department of Livestock and Pasture Science, Faculty of Science and Agriculture, University of Fort Hare, Alice 5700, South Africa; ^2^MPED Research Center, Department of Botany, University of Fort Hare, Alice 5700, South Africa

## Abstract

The global meat industry is characterised by a growing interest in natural preservative additives. This study determined the effect of sweet basil (*Ocimum basilicum L*.) essential oil (SBEO) on colour and lipid oxidation in minced beef. The phytoconstituents of SBEO were analyzed by gas chromatography mass spectrometry. Thereafter, minced beef samples from Nguni and Boran cattle were treated with either no additives (control, C) or SBEO added at 2% (SB2), 4% (SB4), or 6% (SB6). The meat samples were aerobically packaged and stored (4 ± 1°C) for seven days for measurement of lightness (L⁎), redness (a⁎), yellowness (b⁎), hue, chroma, and lipid oxidation (acid-reactive substances, TBARS) on days 0, 4, and 7. Thirty-two bioactive compounds with reported antioxidant and antimicrobial and activities were identified in SBEO, including Estragole (41.40%), 1, 6-Octadien-3-ol, 3,7-dimethyl (29.49%), and trans-.alpha.-Bergamotene (5.32%). On days 0, 4, and 7, SB2, SB4, and SB6 had higher (P < 0.05) L⁎, a⁎, b⁎, hue, and chroma values; and on days 0 and 4 TBARS were lower (P < 0.05) in SB2 and SB4 than C and SB6. The addition of 2% and 4% SBEO improved colour and lipid oxidative stability, demonstrating potential for its use as a natural antioxidant additive in meat.

## 1. Introduction

Globally, the use of antioxidants as preservatives has been instrumental in improving the quality and extending the shelf life of muscle foods, especially during processing and storage [1]. This is because meat and meat products are easily susceptible to oxidation and microbial contamination due to their chemical composition and rapid depletion of endogenous antioxidants postmortem [2]. Reports have shown that live muscle contains relative amount of endogenous antioxidants, including alpha-tocopherol, histidine-containing dipeptides, ubiquinone, glutathione, carnosine, and anserine, which are capable of scavenging free radicals and disrupting oxidative process* in vivo* [2–4]. However, after slaughtering, this muscle tissue begins to lose its antioxidative potential due to various postslaughter conditions such as anaerobic environment, presence of free radicals (reactive oxygen and nitrogen species), and lack of enzymatic mechanisms [5, 6].

As postmortem time increases, the activities of these endogenous antioxidants continue to diminish [2, 6], thereby exposing the lipid and protein component of muscle to rapid deterioration. The rate at which the endogenous antioxidants decrease during postmortem may depend on animal species, breed (including heme pigments), nutrition, muscle part, antemortem stress, and physiological functions [7, 8]. In an attempt to boost meat antioxidant content, different antioxidants (natural or synthetic) are used in the meat industry. Recently, the application of synthetic antioxidant in food/meat products has been implicated in causing negative health effect on consumers [6, 9, 10], thereby promoting interest in the utilization of natural antioxidants. Antioxidants are abundantly found in a wide range of natural sources including fruits, herbs grains, spices, nuts, seeds, leaves, and roots [6].

Sweet basil (*Ocimum basilicum L*.) is one of the frequently used culinary herbs (family of Lamiaceae), known to possess strong antioxidant and antimicrobial activities due to its phenolic acids and aromatic compounds [9, 11]. The plant grows annually or perennially in Asia, India, Africa, and other temperate climate regions throughout the world [9]. Traditionally, every part of the plant is used as medicine to treat headaches, coughs, diarrhea, constipation, warts, worms, kidney malfunction, and digestive problems [12]. According to Marwat et al. [9], basil plant has moderate macro and micro nutritional values including protein (3.15 g/100 g), fat (0.64 g/100 g), energy (23 Kcal), Vitamin C (18 mg/100 g), Vitamin E (0.80 mg/100 g), Vitamin A (5275 IU), Vitamin K (414.8 mcg), Calcium (177 mg/100 g), Iron (3.17 mg/ 100g), Potassium (295 mg/100 g), Magnesium (64 mg/100g), and Sodium (4 mg/100 g).

The* in vitro* activity of its essential oils has been reported to exhibit antimicrobial, antifungal, anticancer, anticonvulsant, hypnotic, and antioxidant activities [13, 14]. The inclusion of basil leaf extract in minced pork at 0.3 g/kg has been reported to lower the microbial population, oxidative deterioration, and improve the sensory quality compared to the control after 5 days of cold storage at 1°C [15]. However, information on the antioxidant effect of sweet basil essential oil in meat products is rarely available. The consumption of meat products containing antioxidant-rich herbs and spices has been reported to reduce* in vivo* formation of malondialdehyde and lower the risk of cancer and cardiovascular disease [16]. Therefore, this study was designed to investigate the preservative effect of sweet basil essential oil on physicochemical characteristics and lipid oxidation of minced beef during cold storage.

## 2. Materials and Methods

### 2.1. Collection of Essential Oil and GC-MS Analysis

Organic essential oil from sweet basil leaf, extracted by hydrodistillation, was obtained (Faithful to Nature, South Africa). The phytoconstituents of the oil were analyzed by gas chromatography mass spectrometry (GC-MS). The GC-MS analysis of essential oils was quantitatively performed using an Agilent 7890B GC system coupled with an Agilent 5977A, Chemetrix (pty) Ltd.; Agilent Technologies, DE (Germany) with a Zebron-5MS column (ZB-5MS 30 m × 0.25 mm × 0.25 *μ*m) (5%-phenylmethylpolysiloxane). GC-grade helium was used as carrier gas at a constant flow rate of 2mL/min and splitless was also used at 1ml injection. The injector, source, and oven temperature were set at 280°C, 280°C and 70°C, respectively. The ramp setting was initially programmed at 15°C/min to 120°C, then 10°C/min to 180°C, and then 20°C/min to 270°C and held for 3 min. The identification of the chemical constituents of the essential oil was determined by their GC retention time, percentage composition (area %), and retention indices. The Kovat indices were calculated according to a set of standard hydrocarbons (C9-C20) [17]. The interpretation and identification of their mass spectra were confirmed by mass spectral incorporated library. Likewise, the retention indices were calculated in line with a homologous series of n-alkanes (C8–C32) under similar operating conditions by using a standard equation. Further identification of the components was made based on computer matching of the mass spectra with the National Institute of Standards and Technology (NIST) database (NIST/EPA/NIH) mass spectral library 2014) with those of published data [18]. Empirical searches were conducted using the PubChem Project (https://pubchem.ncbi.nlm.nih.gov/) and DrugBank (www.drugbank.ca/) to identify the known pharmacological properties associated with these compounds components.

### 2.2. Collection and Preparation of Meat Samples

Fresh beef samples (*longissimus thoracis et lumborum* muscle) were obtained from Boran and Nguni cattle reared on a natural grazing pasture and slaughtered at the age of 18 months with an average live weight of 380 and 260 kg, respectively, in high throughput commercial abattoir (East London, Eastern Cape Province, South Africa). The meat samples from each breed were cut into small cubes after removal of visible fat and connective tissues and minced in a sterile meat grinder. A portion (500 g) from the ground meat was randomly assigned to one of the following treatments: (1) C (control, meat without additives); (2) SB2 (meat with 2% sweet basil essential oil); (3) SB4 (meat with 4% sweet basil essential oil); and (4) SB6 (meat with 6% sweet basil essential oil); with four replicates per treatment. Immediately after adding the essential oil, the ground meat samples were aerobically packed in polyethylene bags (O2 permeability = 6000–8000 cm3/(24 h × m2 × atm), water vapour transmission = 83 g/ (24 h × m2) and 50% relative humidity), stored at 4 ± 1°C, and analyzed on 0, 2, 4, and 7 days of storage for colour and thiobarbituric acid-reactive substances (TBARS).

### 2.3. Physicochemical Characteristics

#### 2.3.1. Instrumental Colour Determination

Colour changes in fresh meat during storage were performed using Hunter Lab Minolta colorimeter (BYK-Gardener GmbH, USA) with 20 mm aperture set for illumination D65 at 100 standard observer angles. The CIE colour coordinates L*∗* (lightness), a*∗* (redness), and b*∗* (yellowness) were measured perpendicular to the meat surface at three different points after calibration using the standard green, black, and white colour samples. All the colour parameters (L*∗*, a*∗*, and b*∗*) were obtained from the mean of readings taken from four replicates per treatment. Hue (an indicator of the angle at which a vector radiates into the red-yellow quadrant) and chroma (a measure of colour saturation) were calculated as follows [19]:

(1) Hue  angle=tan-1b∗a∗Chroma=a∗2+b∗21/2

#### 2.3.2. Determination of Lipid Oxidation (TBARS)

The lipid oxidation of the ground beef was measured using TBARS, by a modified acid precipitation method. Two grams of each sample was weighed in triplicate into 50 mL tubes, 6.25 mL trichloroacetic acid (TCA, 0.001M) and 6.25 mL distilled water (dH2O) were added, and samples were homogenised (Ultraturax) for 20 sec. Slurry was left to filter through a Wattman no1 filter paper. From a stock solution of 1,1,3,3-Tetramethoxypropan (TMP, 0.001M), a standard curve was prepared in duplicate by adding 0, 5, 10, and 20 *μ*L TMP in 1 mL of dH2O. Three tubes were allocated to each sample and 1 mL of filtered slurry was added to each tube. One millilitre of TBA was added to each standard and to 2 tubes for each sample, while 1 mL of dH2O was added to the third sample tube to act as a turbidity blank. All tubes were capped, vortexed, and incubated in a water bath at 70°C for 1 h. Thereafter, samples were allowed to cool, 200 *μ*L, and the absorbance was read at 530 nm. TBARS, expressed as mg of malondialdehyde (MDA)/kg meat, were calculated as

(2) $$\begin{array}{c} TBARS\;\left(\;mg\;\;MDA/Kg\;\;meat\;\right)=\frac{absorbance*molar\;\;mass\;\;of\;\;MDA*volume\;\;of\;\;extract*dilution\;\;factor}{sample\;\;mass*slope\;\;of\;\;standard\;\;curve} \end{array}$$

All TBARS analysis was carried out on four replicates per each treatment and storage day.

## 3. Statistical Analysis

Data obtained on antioxidant contents of the sweet basil essential oil were analyzed using PROC ANOVA procedures of the Statistical Analysis System (SAS, version 9.1.3 of 2007). The colour and TBARS values were analyzed using PROC GLM procedures of SAS (version 9.1.3 of 2007). Significant differences between the least square means for meat samples were performed using the Fishers' least significance difference (LSD) method of SAS, with significance level of p < 0.05.

## 4. Results and Discussion

### 4.1. Chemical Constituent of the Essential Oil of* Ocimum basilicum L*.

**Figure 1:**
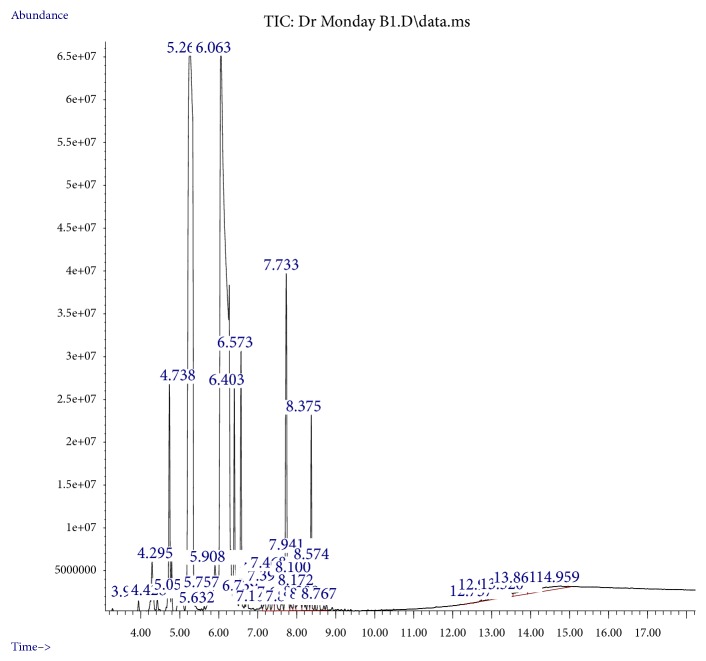
GC-MS chromatogram of sweet basil essential oil.

The chemical composition of the sweet basil essential oils is presented in Table 1. The GC-MS analysis of the oil indicated a total of thirty-two (32) individual compounds (Figure 1), along with their retention indices, retention time, and percentage of composition. Our result demonstrated that the main constituents in the essential oil were Estragole (41.40%), 1,6-Octadien-3-ol, 3,7-dimethyl (29.49%), trans-.alpha.-Bergamotene (5.32%), Eucalyptol (3.51), Citral (3.31%), N-Cyano-3-methylbut-2-enamine (3.08%), cis-.alpha.-Bisabolene (1.92%), Levomenthol (1.81%), and beta.-Myrcene (1.11%). Other important constituents identified in the oil with evidence of biological activities as antioxidant and antimicrobial were alpha.-Pinene (0.13%), cis-Linaloloxide (0.75%), Eugenol (0.40%), Copaene (0.38%), Humulene (0.59%), and Nerolidol (0.08%) (Table 2). The amount of compounds detected from this study equalled those reported by Złotek et al. [20], Al Abbasy et al. [21], and Okoye et al. [22] but were slightly lower than those reported by Widyawati et al. [23] and Tsasi et al. [12]. This variation could be attributed to physiological status, climate change, geographic location, harvesting time, mode, and method of extraction [24, 25]. The presence of essential oils and their composition determines the specific aroma and colour of plant and also an array of flavors when consumed [26].

### 4.2. Physicochemical Characteristics of Meat

Meat colour is one of the most important parameters that influence consumer's decision in buying, selecting, and acceptability of meat and meat products during display [27]. The addition of sweet basil essential oil improved (P < 0.05) the colour of the meat samples compared to control. The results revealed a significant breed effect in meat colour parameters (lightness (L*∗*), yellowness (b*∗*), redness (a*∗*), and chroma and hue values in the meat samples across the storage days (Table 3)).

In Table 3, L*∗*- and b*∗*- values varied significantly among treatments and storage period. From days 0 to 7, all the meat samples treated with basil EO reflected the higher L*∗* and b*∗* values than control group. This result is in agreement with the findings of Ünal et al. [28] and Karabagias et al. [29] who reported that meat treated with oregano, sage, rosemary, and thyme essential oil essential oils had higher L*∗* and b*∗* values than the control group throughout the storage period. This is probably due to the protective effect of essential oil treatments on colour lightness.

The result of the a-value of the beef samples in this study are shown in Table 3, with the value progressively decreasing across the days of storage within the treatments. This is in agreement with other studies who have reported remarkable decrease in redness intensity of meat samples (beef, mutton, and chicken) treated with essential oil during cold storage period [28, 30, 31]. At day 0, meat sample treated with 6% sweet basil EO had the highest redness values (19.18), followed by sample containing 4% (18.71) and 2 (18.45) sweet basil EO and least in control (17.89). Similar trend was also observed in day 4, with meat sample treated with sweet basil EO showing intense red colour (12.77), higher than the control group (11.44) and other treatments. Ünal et al. [28] in their study also found higher a-value in fresh minced beef treated with essential oil compared to control. However, at the 7, meat sample treated with 2% basil EO had the highest redness (11.30), followed by the control group and least in sample treated with 4 and 6% basil EO. This reduction in redness colour intensity of beef samples treated with 4 and 6% sweet basil EO at day 7 compared to control could be due to self-oxidation of basil EO to now act as prooxidants to increase pigment oxidation. Reports have revealed that, at high concentration, essential oil can behave as prooxidant, damaging cellular biomolecules [32, 33].

Increase in oxidation is strongly related to decrease in a-values of meat products or denaturation the myoglobin molecules thereby negatively reducing the colour of the meat product during cold storage and display [28, 34, 35]. In overall, fresh ground meat from Boran cattle breed exhibited higher colour stability than Nguni cattle breed. This could be due to differences in their genotypic characteristic. Lynch et al. [36] had earlier reported a significant difference in colour of beef from three breeds during storage period.

### 4.3. Lipid Oxidative Characteristics of Meat

The results of the oxidative changes (TBARS) of samples treated with basil essential oil aregiven in Table 4. The findings show that adding sweet basil essential oil can protect ground beef against lipid oxidation. In comparison to control group, meat samples containing sweet basil EO at 2 and 4% had lower the TBARS values from day 0 to 4. The inhibitory effects of the sweet basil essential oil against the TBAS formation could be attributed to the inherent phenolic content, phytoconstituents and antioxidant activity. Many studies have reported a positive correlation between phytochemical content or antioxidant activity of plant essential oil and reduction in lipid oxidation in meat products [15, 28]. The antioxidant activity of phytochemical compounds in essential oil has been associated with the hydroxyl group linked to the aromatic ring, which is capable of donating hydrogen atoms with electrons and neutralizing free radicals [26].

At day 7 of storage meat sample treated with higher concentration (4 and 6% sweet basil EO) reflected higher TBARs values than the control group. This result is similar to the finding of Kuzelov et al. [15] who reported that meat sample treated higher concentration of holy basil extract above 3 mg/kg exhibited higher TBARS values than the control after 5^th^ of cold storage at 1°C. In general, the addition of* sweet basil essential oil* exhibited higher antioxidant activity at 2% than the control and those treated with 4 and 6% essential oil at day 7 of storage.

In overall, the TBARS values of the Boran beef were slightly lower than Nguni beef samples across the treatment and storage period. Since both animals were raised on natural pasture, this difference in their TBARS values could be attributed to factors such as inherent endogenous antioxidants, and composition and distribution of unsaturated fatty acids in triacylglycerol molecule, which has been reported to influence the rate of lipid oxidation in muscle food [37]. This result is in agreement with the report of Xie et al. [38] who found that Limousin beef sample has significant lower TBARS values than Qinchuan cattle breed.

## 5. Conclusion

A total of 32 bioactive compounds were identified in SBEO, with reported preservative functions including antioxidant and antimicrobial activities, as well as anti-inflammatory, nematicidal, and anticancer compounds. The addition of 2% and 4% SBEO improved colour stability of minced beef during 7 days of refrigerated storage compared to the control. The TBARS were significantly lower in SB2 and SB4 on days 0 and 4, while SB6 was similar to or higher than C. The results indicate the potential of SBEO at 2 and 4% as a natural antioxidant additive to improve colour and lipid oxidative stability during refrigerated storage of aerobically packaged minced beef, and further studies investigating its antimicrobial activity are recommended.

## Figures and Tables

**Table 1:** Chemical composition of *sweet basil* leaf essential oil.

No	Compounds	Retention indices	Formula	Retention Time (min)	% Peak Area
1	alpha.-Pinene	935	C_10_H_16_	3.946	0.13
2	beta.-Myrcene	991	C_10_H_16_	4.295	1.11
3	4-Hexen-1-ol, acetate	857	C_8_H_14_O_2_	4.428	0.22
4	Eucalyptol	1033	C_10_H_18_O	4.738	3.51
5	cis-Linaloloxide	1090	C_10_H_18_O_2_	5.055	0.75
6	1,6-Octadien-3-ol, 3,7-dimethyl	1099	C_13_H_22_O_2_	5.261	29.49
7	Methyl ethyl cyclopentene	1113	C_8_H_14_	5.632	0.08
8	l-Menthone	1128	C_10_H_18_O	5.757	0.48
9	Levomenthol	1172	C_10_H_20_O	5.908	1.81
10	Estragole	1206	C_10_H_12_O	6.063	41.40
11	N-Cyano-3-methylbut-2-enamine	1238	C_6_H_10_N_2_	6.403	3.08
12	Citral	1270	C_10_H_16_O	6.573	3.13
13	Cyclohexene, 4-methyl-1-(1-methyle thyl)	977.5	C_10_H_18_	6.739	0.42
14	Phenol, 2,3,5-trimethyl	1492	C_9_H_12_O	7.101	0.18
15	Eugenol	1358	C_10_H_12_O_2_	7.185	0.40
16	Formic acid, cyclohexyl ester	1304	C_7_H_12_O_2_	7.300	0.40
17	Copaene	1495	C_15_H_24_	7.395	0.38
18	cis-7,10,13,16-Docosatetraenoic acid, methyl ester	1393	C_23_H_38_O_2_	7.468	0.55
19	Neoisolongifolene	1411	C_15_H_24_	7.635	0.20
20	trans-.alpha.-Bergamotene	1433	C_15_H_24_	7.733	5.32
21	Alloaromadendrene	1452	C_15_H_24_	7.852	0.12
22	Humulene	1432	C_15_H_24_	7.941	0.59
23	beta.-copaene	1477	C_15_H_24_	8.100	0.71
24	beta.-Bisabolene	1509	C_15_H_24_	8.172	0.38
25	cis-muurola-3,5-diene	1502	C_15_H_24_	8.309	0.18
26	cis-.alpha.-Bisabolene	1504	C_15_H_24_	8.375	1.92
27	Nerolidol	1535	C_15_H_26_O	8.471	0.08
28	trans-4-Methoxycinnamaldehyde	1569.8	C_10_H_10_O_2_	8.574	0.71
29	Benzeneacetic acid,.alpha.-hydrox	1517	‎C_8_H_8_O_3_	8.767	0.06
30	Phenylethanolamine	1298	C_8_H_11_NO	8.767	0.09
31	3-Methyl-2-phenylindole	1710	C_15_H_13_N	12.737	0.01
32	N-Benzyl-N-ethyl-p-isopropylbenzamide	1973	C_19_H_23_NO	13.520	0.38

**Table 2:** Bioactivity of phytocomponents identified in the essential oil of *Sweet basil* by GC-MS.

**No**	**Compound**	**Compound Structure**	**Molecular Weight (g/mol)**	**Biological activities**
**1**	alpha.-Pinene	C_10_H_16_	136.24	antimicrobial
**2**	beta.-Myrcene	C_10_H_16_	136,23	Antioxidant, antimicrobial
**3**	Eucalyptol	C_10_H_18_O	‎ 154.25	Antimicrobial
**4**	cis-Linaloloxide	C_10_H_18_O_2_	170.25	Nematicidal
**5**	Levomenthol	‎C_10_H_20_O	156.27	antimicrobial
**6**	Citral	C_10_H_16_O	152.24	Antioxidant, antimicrobial
**7**	Eugenol	C_10_H_12_O_2_	164.20	Antioxidants, antimicrobial
**8**	Copaene	C_15_H_24_	204,36	Antioxidant
**9**	Humulene	C_15_H_24_	204.36	Anti-inflammatory
**10**	Nerolidol	C_15_H_26_O	222.37	Antioxidant, anticancer, antimicrobial
**11**	Estragole	C_10_H_12_O	148.2	Antimicrobial, anti-inflammatory,

**Table 3:** Preservative effect of sweet basil essential oil on colour stability of minced beef from Boran and Nguni cattle during cold storage at 4°C.

**Parameters**	**Days**	**Treatment (T)**				**Breed (B)**		**SEM**	***P-value***		
		**Control**	**SB2**	**SB4**	**SB6**	**Boran**	**Nguni**		**T**	**B**	**B x T**
**Lightness (L)**	0	34.93^d^	34.39^c^	35.51^b^	36.64^a^	34.92^b^	35.82^a^	0.56	0.01	0.03	0.04
	4	37.88^a^	38.13^a^	38.49^a^	38.18^a^	37.58^a^	38.72^a^	0.89	0.89	0.07	0.78
	7	35.18^c^	37.38^b^	37.82^b^	39.38^a^	36.78^b^	38.09^a^	0.40	<0.01	<0.01	<0.01

**Yellowness (b)**	0	16.43^b^	16.32^b^	16.91^ab^	17.42^a^	16.52^b^	17.02^a^	0.31	0.03	0.003	0.33
	4	12.03^c^	13.16^b^	13.96^a^	14.25^a^	12.91^b^	13.79^a^	0.20	<0.01	<0.01	0.01
	7	9.97^c^	12.04^b^	12.33^b^	13.87^a^	11.79^b^	12.32^a^	0.30	0.01	<0.02	0.04

**Redness (a)**	0	17.89^c^	18.45^b^	18.71^b^	19.18^a^	18.80^a^	18.32^a^	0.34	0.01	0.54	0.01
	**4**	11.44^d^	11.53^c^	12.15^b^	12.77^a^	11.72^b^	12.22^a^	0.40	0.01	<0.01	0.001
	7	10.83^b^	11.30^a^	10.39^b^	10.82^b^	11.08^a^	10.58^b^	0.37	0.05	0.03	0.15

**Chroma**	0	24.30^c^	24.65^bc^	35.24^a^	25.91^b^	25.05^a^	25.02^a^	0.43	0.01	0.91	0.03
	4	16.73^d^	17.50^c^	18.52^b^	19.14^a^	17.45^b^	18.43^a^	0.31	<0.01	<0.01	0.05
	7	14.73^c^	16.55^b^	16.23^b^	17.62^a^	16.32^a^	16.25^a^	0.32	<0.01	0.16	0.12

**Hue**	0	0.74^a^	0.73^a^	0.74^a^	0.74^a^	0.72^b^	0.75^a^	0.00	0.14	0.01	0.01
	4	0.83^a^	0.85^a^	0.85^a^	0.84^a^	0.84^a^	0.85^a^	0.01	0.32	0.72	0.01
	7	0.73^c^	0.81^b^	0.88^a^	0.90^a^	0.80^b^	0.85^a^	0.02	<0.01	<0.01	<0.01

C: no additives; SB2: 2% sweet basil essential oil; SB4: 4% sweet basil essential oil; SB6: 6% sweet basil essential oil.

Means not sharing a common superscript (with ^a-b^) in a row for each treatment are significantly different at p < 0.05.

**Table 4:** Preservative effect of sweet basil essential oil on oxidative stability of minced beef from Boran and Nguni cattle during cold storage at 4°C.

**Days**	**Treatment (T)**				**Breed (B)**		***P-value***		
	**Control**	**SB2**	**SB4**	**SB6**	**Boran**	**Nguni**	**T**	**B**	**B x T**
**0**	0.60±0.31	0.19±0.28	0.22±0.28	0.65±0.24	0.31±0.20	0.53±0.19	0.66	0.46	0.04
**4**	1.03±0.09	0.90±0.11	1.00±0.09	1.13±0.10	0.90±0.07	1.14±0.06	0.77	0.42	0.78
**7**	1.00±0.09	0.96±0.08	1.11±0.11	1.24±0.08	0.96±0.06	1.19±0.07	0.17	0.15	<0.01

## Data Availability

The data used to support the findings of this study are available from the corresponding author upon request.
